# Persistent Stapedial Artery in Adhesive Otitis Media: A Case Report

**DOI:** 10.7759/cureus.66922

**Published:** 2024-08-15

**Authors:** Norah S AlOtaibi, Sarah S AlOtaibi, Abdulaziz M AlAbdulkareem, Naif K AlOsaimi, Faisal A AlFadhel, Fahad N AlTamimi

**Affiliations:** 1 College of Medicine, Alfaisal University, Riyadh, SAU; 2 Department of Otolaryngology, Head and Neck Surgery, King Faisal Specialist Hospital and Research Centre, Riyadh, SAU; 3 Department of Otolaryngology, Head and Neck Surgery, King Saud Medical City, Riyadh, SAU; 4 Department of Otolaryngology, Head and Neck Surgery, Ministry of National Guard Health Affairs, Riyadh, SAU; 5 Department of Radiology, King Faisal Specialist Hospital and Research Centre, Riyadh, SAU

**Keywords:** widened facial nerve canal, congenital vascular anomaly, absent foramen spinosum, conductive hearing loss, pulsatile tinnitus, adhesive otitis media, persistent stapedial artery

## Abstract

A persistent stapedial artery (PSA) is a rare embryologic remnant that typically involutes at week 10 of embryogenesis. However, if it is persistent, it may lead to conductive hearing loss and pulsatile tinnitus. It is of utmost importance to identify such an anomaly, as it leads to serious complications intraoperatively if overlooked. Proper clinical and radiological assessment helps an otologist recognize the PSA. We describe the case of a 24-year-old female presenting with a chronically discharging ear in addition to pulsatile tinnitus and conductive hearing loss with an incidental finding of a PSA upon otoscopy.

## Introduction

The persistent stapedial artery (PSA) is a rare congenital vascular anomaly, originating from the second aortic arch, with a prevalence of 0.02-0.48% [1-3]. The stapedial artery (SA), which connects the branches of the future external carotid artery (ECA) to the internal carotid artery (ICA), is transiently present during normal fetal development [1]. Rarely does the SA persist after embryogenesis [1].

Patients with a PSA are usually asymptomatic, and it is typically incidentally found during middle ear surgery [1,4,5]. However, patients may present with conductive hearing loss, pulsatile tinnitus, or a pulsating middle ear mass [1,4,6]. 

Temporal bone imaging, particularly on computed tomography (CT), is usually enough to identify a PSA. A widened fallopian canal and an absent ipsilateral foramen spinosum are two classic CT findings that suggest a PSA [1,4,5]. Since helically acquired source images are used for almost all routine head CT scans, resolutions of 0.75 or 0.625 mm may be sufficient to visualize the PSA above the absent foramen spinosum [4]. Furthermore, a PSA on CT should prompt additional investigations for possible concurrent imaging malformations, as it strongly correlates with other congenital anomalies [4]. Temporal bone imaging helps identify the PSA to prevent unnecessary surgery and plan endovascular or surgical interventions [1]. 

Middle ear tissues may become adherent in adhesive otitis media, a chronic suppurative otitis media caused by ongoing inflammation [7-9]. It is among the most common otolaryngology conditions [7-9]. Adhesive otitis media patients often present with complete or partial adhesions between the medial wall of the middle ear and the thin, atrophic, retracted pars tensa [7-9]. Moreover, soft tissue debris may surround the middle ear ossicles [7-9]. In this study, we present a case of a PSA in adhesive otitis media.

## Case presentation

A 24-year-old non-syndromic female, known to have bilateral cleft lip and palate, presented to our otology service with a history of bilateral chronically discharging ears. She had no other comorbidities. Her surgical history was significant for multiple cleft lip and palate repairs in addition to having pressure equalization tubes inserted twice in childhood. 

She presented with a complaint of gradually progressive decreased hearing bilaterally and had a history of repeated ear infections in addition to non-malodorous otorrhea bilaterally. She had long-standing right-sided pulsatile tinnitus with no associated otalgia, vertigo, headache, autophony, or history of facial weakness.

**Figure 1:**
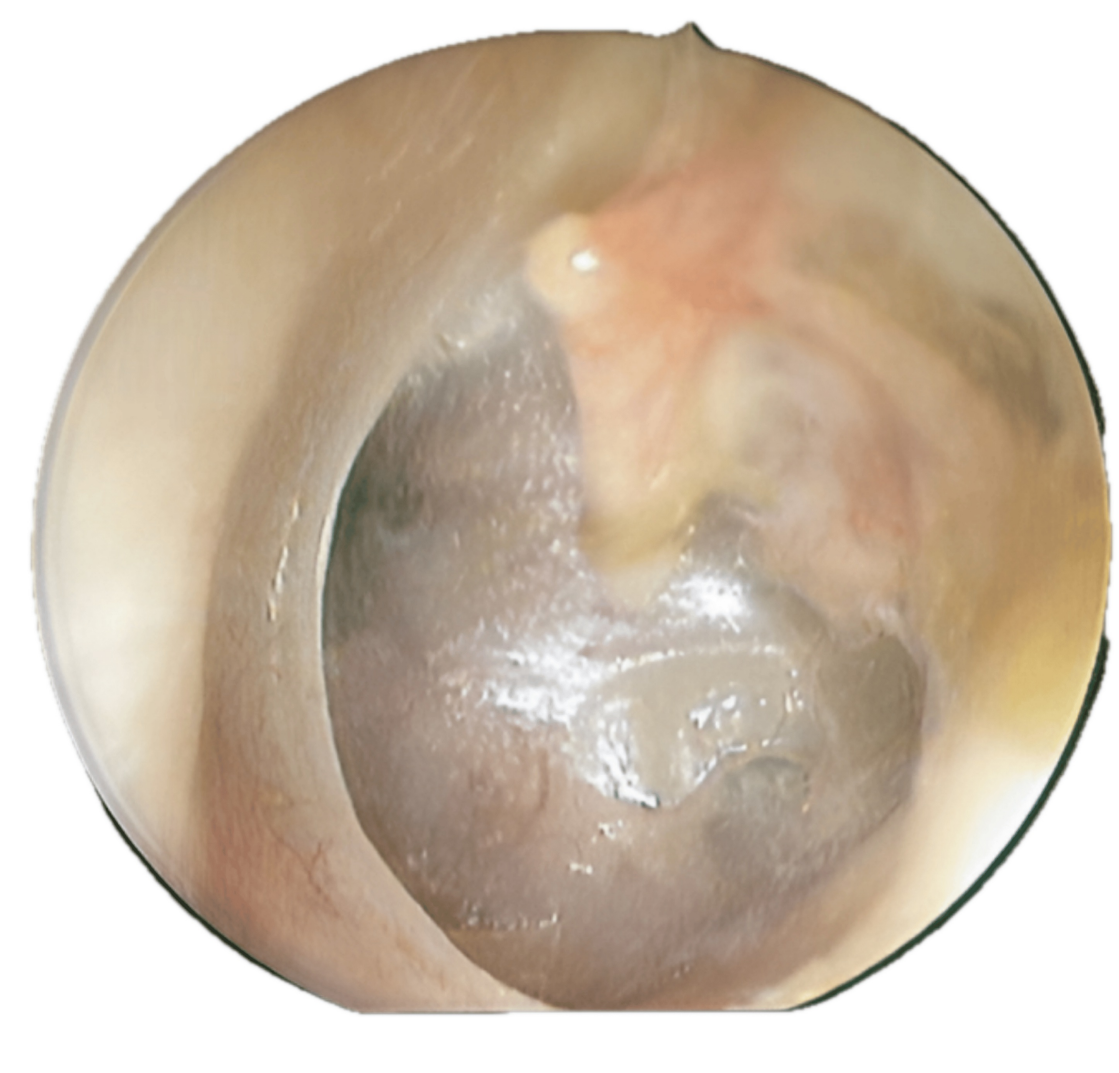
Otoendoscopy of the left ear demonstrates adhesive grade III tympanic membrane retraction

Upon clinical examination, the right ear demonstrated adhesive grade IV tympanic membrane retraction with no active purulent otorrhea. There was no noted granulation tissue or keratin debris. The left ear demonstrated grade III tympanic membrane retraction with no active purulent otorrhea, granulation tissue, or keratin debris (Figure 1). A formal audiological assessment was conducted and revealed a right moderate conductive hearing loss with an average air-bone gap of 40 dB with a type C tympanogram and a left moderately severe mixed hearing loss with a type C tympanogram and normal ear canal volume bilaterally. 

**Figure 2:**
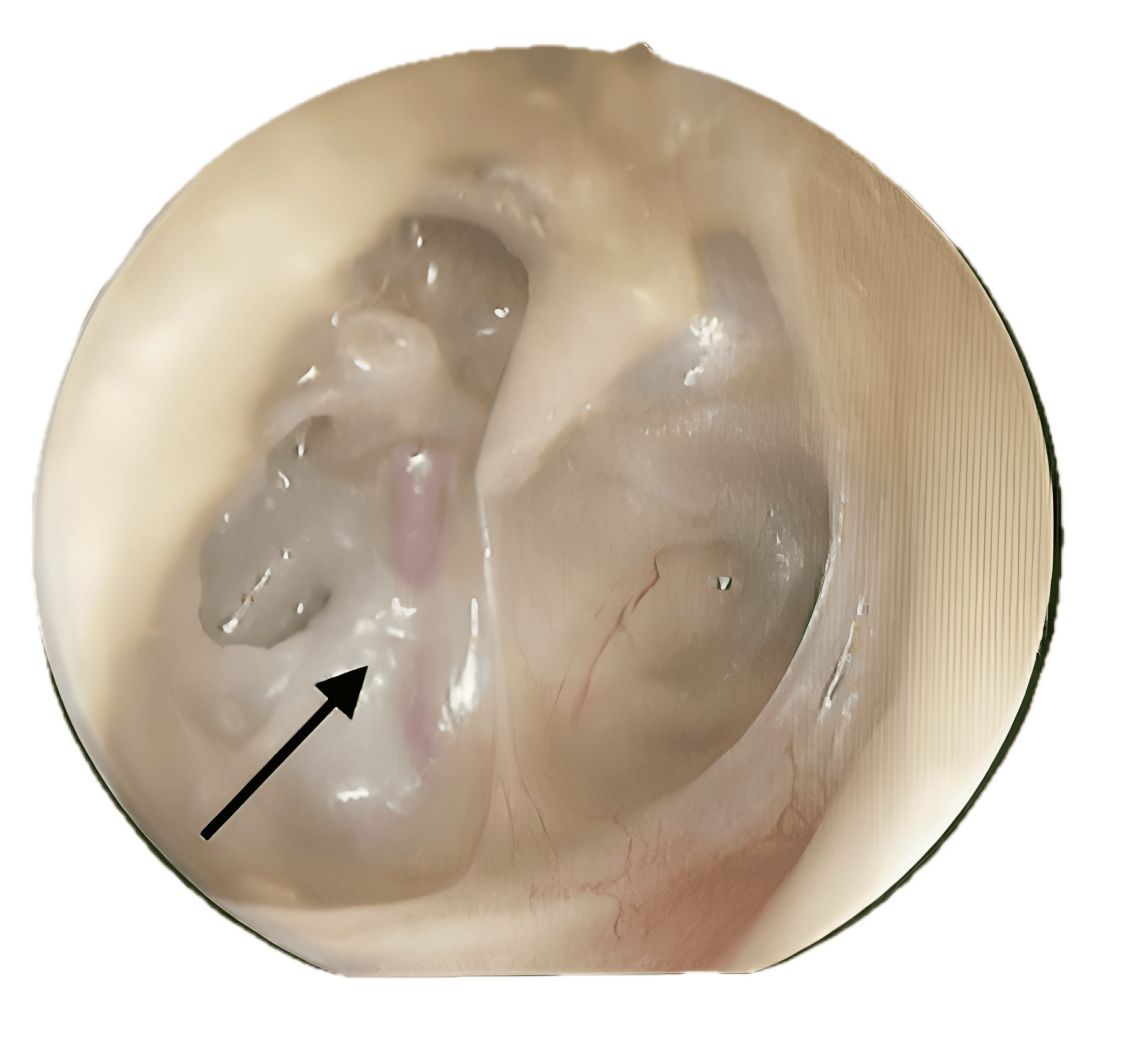
Otoendoscopy of the right ear with adhesive grade IV tympanic membrane retraction and the PSA (black arrow) PSA: persistent stapedial artery

Upon thorough clinical assessment, there was an incidental finding of a right PSA with adhesive grade IV tympanic membrane retraction (Figure 2).

**Figure 3:**
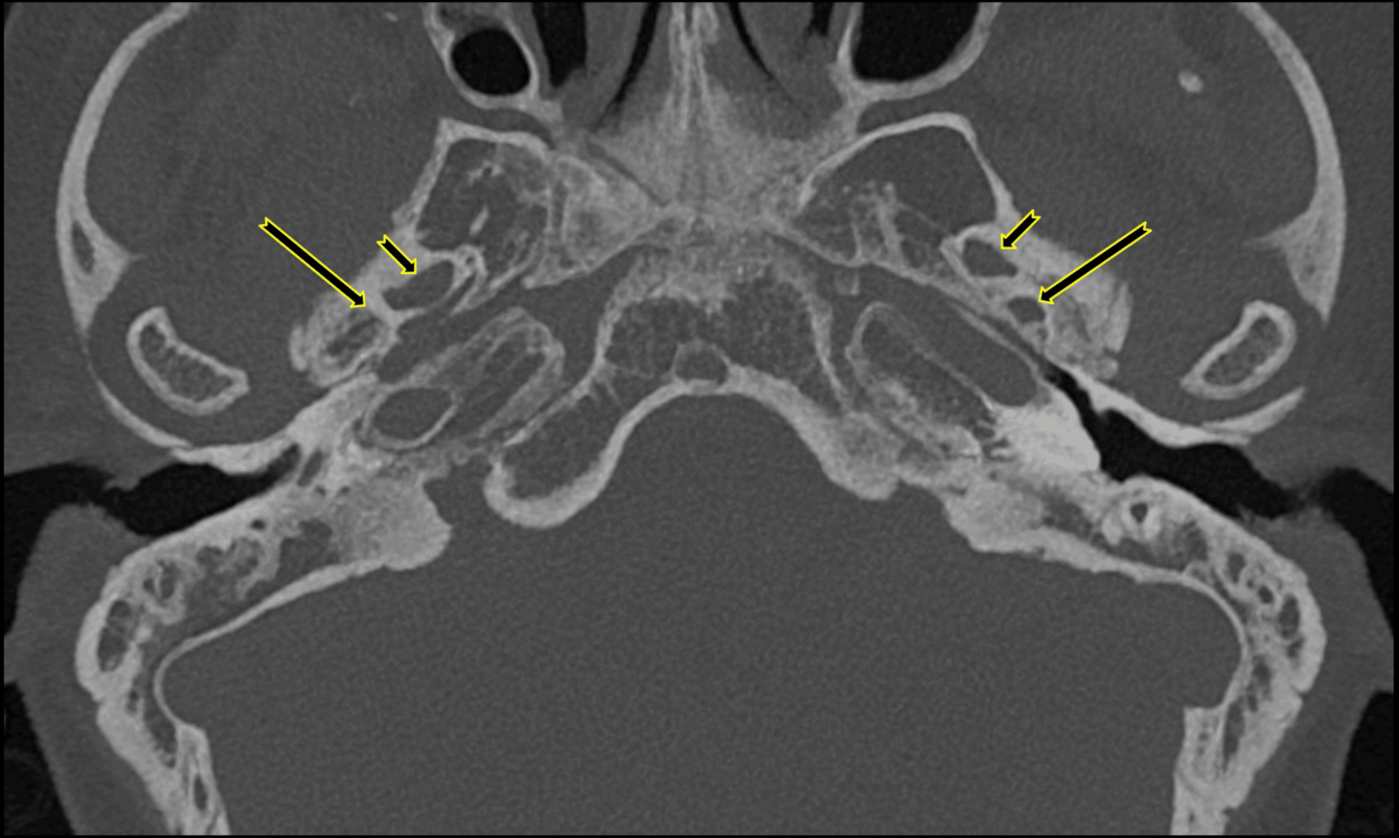
Axial CT reconstructions with maximum intensity projection of the temporal bones with the right PSA Normal foramen spinosum on the left and absent on the right at its expected location (long arrow); note the normal foramen ovale on both sides (short arrow). PSA: persistent stapedial artery

**Figure 4:**
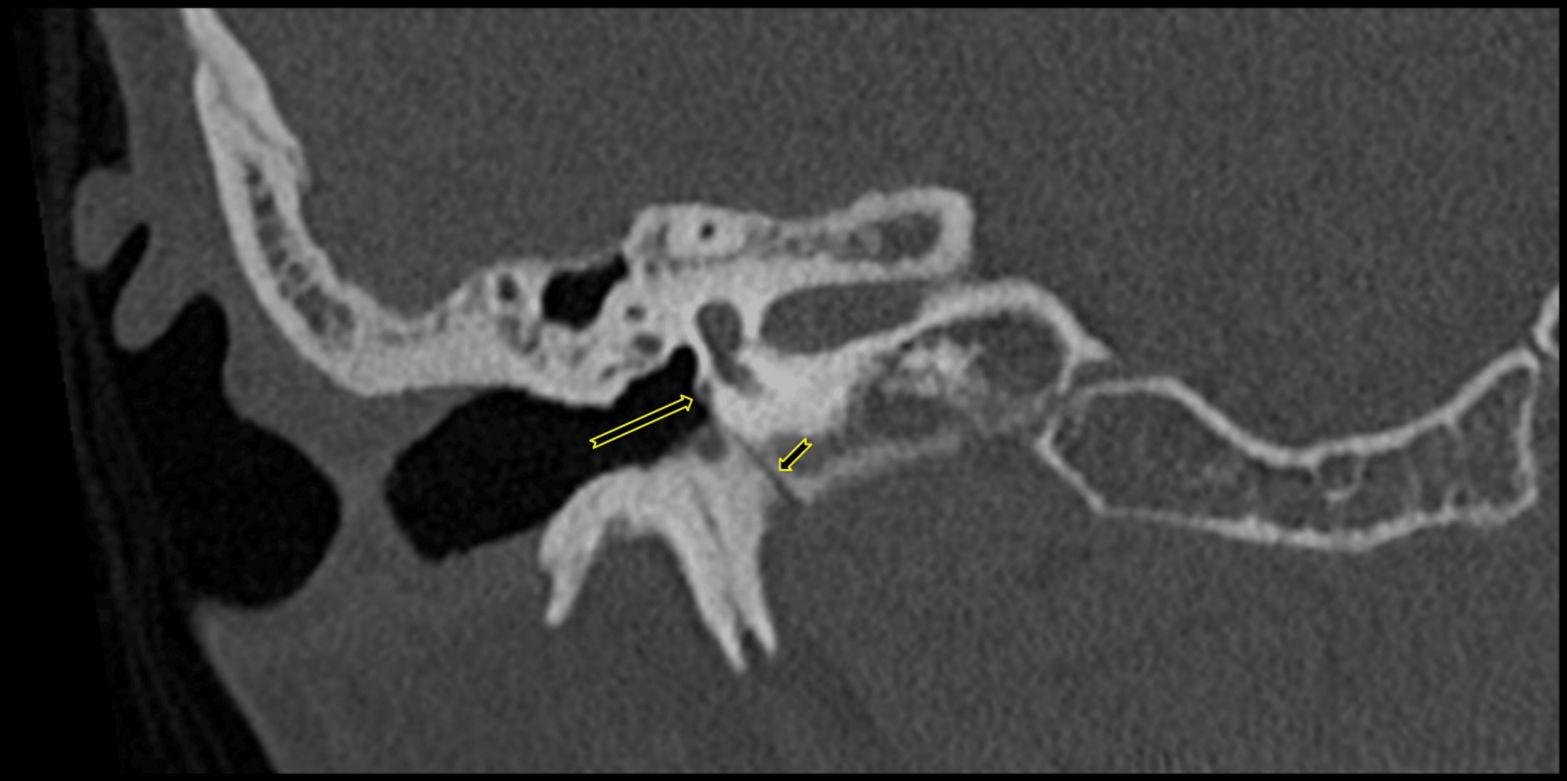
Right temporal bone coronal oblique CT reconstructions demonstrate a small vascular channel of the PSA. A small vascular channel of the PSA (short arrow) leaves the carotid canal, coursing superiorly over the lateral surface of the cochlear promontory (long arrow). PSA: persistent stapedial artery

**Figure 5:**
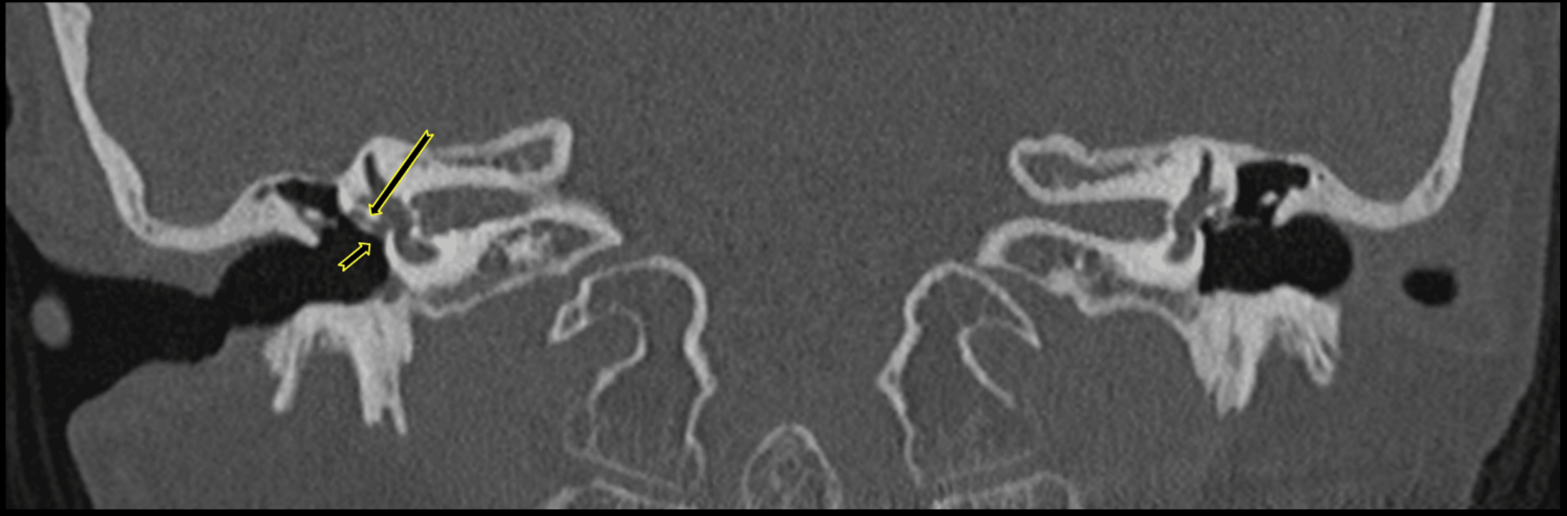
Temporal bone coronal CT reconstructions demonstrate the small vascular channel of the PSA obliterating and crossing the right oval window. The PSA obliterates and crosses the right oval window (short arrow). It passes through a small channel (long arrow) toward the lower infero-medial aspect of the tympanic segment of the facial nerve canal. PSA: persistent stapedial artery

**Figure 6:**
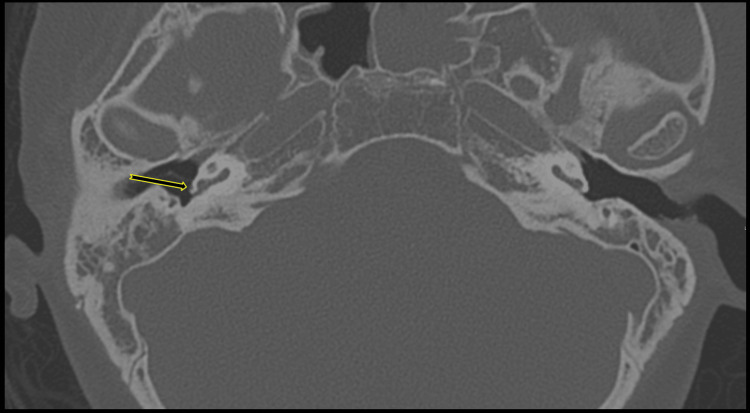
Temporal bone axial CT reconstructions demonstrate the PSA coursing over the right cochlear promontory. A small vascular channel of PSA courses over the lateral surface of the cochlear promontory (long arrow). PSA: persistent stapedial artery

**Figure 7:**
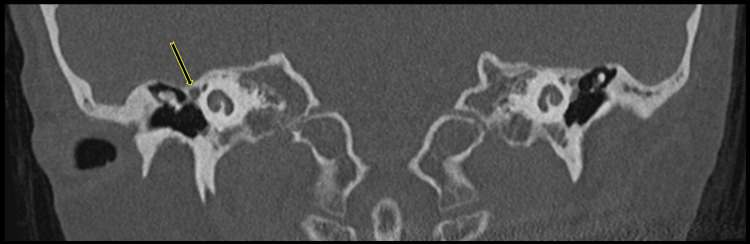
Temporal bone coronal CT reconstructions demonstrate asymmetrical enlargement of the tympanic segment of the right facial nerve canal. Asymmetrical enlargement of the tympanic segment of the right facial nerve canal (long arrow), harboring both PSA and the tympanic segment of the facial nerve. PSA: persistent stapedial artery

A nonenhanced CT of the temporal bone was carried out. It demonstrated a PSA, an absent foramen spinosum, and a widened facial nerve at the geniculate ganglion and tympanic segment. The findings are shown in Figures 3-7.

At that point, the patient was counseled on all treatment options, including serial clinical surveillance, hearing aids, and pressure equalization tubes given the risks. She decided to proceed with hearing aids. 

## Discussion

The SA is an embryologic derivative of the primitive hyoid arch (HA) of the petrous ICA [1,10]. The HA is a dorsal remnant of the second aortic arch that rapidly elongates and gives an anastomosis to the mandibular artery, a remnant of the first aortic arch [10]. The SA extends cranially from the hyoid artery, passes through the mesenchymal primordium of the stapes, and forms the obturator foramen of the stapes [1]. The SA subsequently enters the cranial cavity and divides into the supraorbital and maxillomandibular divisions [1,10]. The SA continues to elongate into the gasserian region, passing into the future tympanic cavity and the crus of the stapes, where it reaches its maximum development [10]. The supraorbital artery gives rise to the middle meningeal artery (MMA) and orbital branches [1,10]. The maxillomandibular artery gives rise to the infraorbital and mandibular branches that develop into the infraorbital and inferior alveolar arteries, respectively [1,10]. They leave the cranial cavity via the foramen spinosum. The ventral pharyngeal artery (VPA), arising from the first and second aortic arches, anastomoses with the maxillomandibular branch and forms the proximal portion of the MMA [1,10]. The VPA later becomes the adult ECA. As flow reverses at the foramen spinosum, the tympanic portion of the SA regresses and leaves behind the caroticotympanic artery and the superior tympanic artery from the HA of the ICA and the petrous branch of the ECA, respectively [1,3,10]. 

Several variants of the PSA have been described in the literature [6,10]. The more frequent variant seen is the partial persistence of the SA, where only the intracranial portion of the SA is present [10]. It ends up giving rise to the MMA [10]. The foramen spinosum is either absent or reduced in size, indicating that a foramen cannot form without its’ contents [10]. The PSA would follow its’ typical course, arising from the petrous ICA, entering the anteromedial hypotympanum, and contained in an osseous canal [1]. In other cases, the PSA may be associated with an aberrant ICA where it follows an intratympanic course [1]. This can be explained by the segmental agenesis of the ICA, where the ascending pharyngeal artery enters the tympanic cavity through Jacobson’s canal [1]. While there, it anastomoses with the caroticotympanic branch of the ICA [1]. The pharyngo-tympano SA is an exceedingly rare variant where the MMA arises from the cervical portion of the ICA [10]. In some settings, the MMA may even arise from the ophthalmic artery [1,10]. 

Most patients with a PSA are reported to be asymptomatic [1,2,4,6]. However, conductive hearing loss and pulsatile tinnitus have also been reported [1,3,4,6], similar to the patient in this study. The PSA is reported to be associated with stapes footplate ankylosis, malformed stapes supra-structure, malpositioned facial nerve, thickened middle ear mucosa that hides the PSA, and oval window atresia [2]. It has also been associated with congenital anomalies such as trisomies 13, 15, and 21, Paget's disease, otosclerosis, thalidomide deformities, anencephaly, congenital immunodeficiency, and neurofibromatosis [1,3,4]. This coincides with our patient, who had bilateral cleft lip and palate. It is imperative to carry out imaging and proper planning before middle ear surgery to prevent accidental injuries to the PSA, and often, at times, surgery may be aborted to avoid catastrophic bleeding [1,6]. The complications of middle ear surgery with a PSA include intraoperative bleeding [4-6], floating footplate [6], and facial nerve palsy [1,4].

The classic imaging features of PSA include an absent foramen spinosum and enlargement of the anterior tympanic segment of the facial nerve canal [1,3-5]. Our patient similarly exhibits those findings on CT. LoVerde et al. also described several characteristic features of the PSA on CT scans [4]. The most exhibited feature in their series is the “string” sign, where the proximal PSA is usually observed as a thin linear soft tissue attenuation crossing over the cochlear promontory after emerging from its’ bony canal seen on sagittal and coronal planes [4]. If a PSA accompanies a duplicated facial nerve canal, then a rounded soft tissue mass lateral to the tympanic portion of the facial nerve is seen in the coronal view of the CT scan [4]. It appears as a “third eye” because the usual two eyes correspond to the labyrinthine and tympanic segments of the facial nerve axially [4]. The PSA at the level of the geniculate ganglion, lateral to the tympanic segment of the facial canal, appears as a tubular soft tissue attenuation moving lateral to the tympanic segment of the facial nerve [4]. It transforms the “V shape” of the facial nerve canal into an “N” shape [4]. Axially, the PSA appears as a discrete dot in the obturator foramen [4]. LoVerde et al. reckon this as a horseshoe encircling a stake, calling it the “ringer” sign [4]. 

## Conclusions

While a PSA is a rare vascular variant, surgeons need to be aware of this phenomenon as it can complicate or even defer middle ear surgery. Even though most patients afflicted by this phenomenon are asymptomatic, it has been reported to cause pulsatile tinnitus and conductive hearing loss. By understanding the embryology and anatomical variants of the SA, surgeons will be better equipped to deal with this anomaly. Imaging studies help to facilitate and mitigate the risks associated with surgery.
